# Increased cancer risk in patients undergoing dialysis: a population-based cohort study in North-Eastern Italy

**DOI:** 10.1186/s12882-019-1283-4

**Published:** 2019-03-28

**Authors:** Martina Taborelli, Federica Toffolutti, Stefania Del Zotto, Elena Clagnan, Lucrezia Furian, Pierluca Piselli, Franco Citterio, Loris Zanier, Giuliano Boscutti, Diego Serraino, for the Italian Transplant & Cancer Cohort Study, Sarah Shalaby, Raffaella Petrara, Patrizia Burra, Giacomo Zanus, Stefano Zanini, Paolo Rigotti, Maria Rendina, Alfredo Di Leo, Francesco Paolo Schena, Giuseppe Grandaliano, Marco Fiorentino, Augusto Lauro, Antonio Daniele Pinna, Paolo Di Gioia, Sara Pellegrini, Chiara Zanfi, Maria Piera Scolari, Sergio Stefoni, Paola Todeschini, Laura Panicali, Chiara Valentini, Umberto Baccarani, Andrea Risaliti, Gian Luigi Adani, Dario Lorenzin, Giuseppe Maria Ettorre, Giovanni Vennarecci, Marco Colasanti, Manuela Coco, Fabrizio Ettorre, Roberto Santoro, Lucia Miglioresi, Francesco Nudo, Massimo Rossi, Gianluca Mennini, Luca Toti, Giuseppe Tisone, Annachiara Casella, Laura Fazzolari, Daniele Sforza, Giuseppe Iaria, Carlo Gazia, Chiara Belardi, Claudia Cimaglia, Alessandro Agresta, Gianpiero D’Offizi, Ubaldo Visco Comandini, Raffaella Lionetti, Marzia Montalbano, Chiara Taibi, Giovanni Fantola, Fausto Zamboni, Gian Benedetto Piredda, Maria Benigna Michittu, Maria Gavina Murgia, Bruno Onano, Lucia Fratino, Luigino Dal Maso, Paolo De Paoli, Diana Verdirosi, Emanuela Vaccher, Francesco Pisani, Antonio Famulari, Federica Delreno, Samuele Iesari, Linda De Luca, Maurizio Iaria, Enzo Capocasale, Elena Cremaschi, Silvio Sandrini, Francesca Valerio, Valentina Mazzucotelli, Nicola Bossini, Gisella Setti, Massimiliano Veroux, Pierfrancesco Veroux, Alessia Giaquinta, Domenico Zerbo, Ghil Busnach, Laura Di Leo, Maria Luisa Perrino, Marialuisa Querques, Valeriana Colombo, Maria Chiara Sghirlanzoni, Piergiorgio Messa, Antonio Leoni, Laura Galatioto, Salvatore Gruttadauria, Vito Sparacino, Flavia Caputo, Barbara Buscemi, Franco Citterio, Gionata Spagnoletti, Maria Paola Salerno, Evaldo Favi, Giuseppe Paolo Segoloni, Luigi Biancone, Antonio Lavacca, Maria Cristina Maresca, Carmelo Cascone, Bice Virgilio, Donato Donati, Fiorella Dossi, Andrea Fontanella, Andrea Ambrosini, Marco Di Cicco

**Affiliations:** 1 Cancer Epidemiology Unit, Centro di Riferimento Oncologico di Aviano (CRO) IRCCS, via Franco Gallini 2, 33081 Aviano, (PN) Italy; 2 Azienda Regionale di Coordinamento per la Salute, Udine, Italy; 3 Kidney and Pancreas Transplantation Unit, Padua University Hospital, Padua, Italy; 4 Department of Epidemiology and Pre-Clinical Research, National Institute for Infectious Diseases “L. Spallanzani”, Rome, Italy; 5 Renal Transplantation Unit, Department of Surgical Science, Università Cattolica Sacro Cuore, Rome, Italy; 6 Azienda Sanitaria Universitaria Integrata di Trieste, Trieste, Italy

**Keywords:** Dialysis, Cancer risk, de novo malignancies, End-stage kidney disease, Population-based study, Italy

## Abstract

**Background:**

In southern Europe, the risk of cancer in patients with end-stage kidney disease receiving dialysis has not been well quantified. The aim of this study was to assess the overall pattern of risk for de novo malignancies (DNMs) among dialysis patients in the Friuli Venezia Giulia region, north-eastern Italy.

**Methods:**

A population-based cohort study among 3407 dialysis patients was conducted through a record linkage between local healthcare databases and the cancer registry (1998–2013). Person-years (PYs) were calculated from 30 days after the date of first dialysis to the date of DNM diagnosis, kidney transplant, death, last follow-up or December 31, 2013, whichever came first. The risk of DNM, as compared to the general population, was estimated using standardized incidence ratios (SIRs) and 95% confidence intervals (CIs).

**Results:**

During 10,798 PYs, 357 DNMs were diagnosed in 330 dialysis patients. A higher than expected risk of 1.3-fold was found for all DNMs combined (95% CI: 1.15–1.43). The risk was particularly high in younger dialysis patients (SIR = 1.88, 95% CI: 1.42–2.45 for age 40–59 years), and it decreased with age. Moreover, significantly increased DNM risks emerged during the first 3 years since dialysis initiation, especially within the first year (SIR = 8.52, 95% CI: 6.89–10.41). Elevated excess risks were observed for kidney (SIR = 3.18; 95% CI: 2.06–4.69), skin non-melanoma (SIR = 1.81, 95% CI: 1.46–2.22), oral cavity (SIR = 2.42, 95% CI: 1.36–4.00), and Kaposi’s sarcoma (SIR = 10.29, 95% CI: 1.25–37.16).

**Conclusions:**

The elevated risk for DNM herein documented suggest the need to implement a targeted approach to cancer prevention and control in dialysis patients.

## Background

Patients receiving renal replacement therapies (RRT) are known to be at higher risk of cancer than the corresponding general population [1–3]. Although some malignancies diagnosed in dialysis patients or after kidney transplant (KT) share similar risk factors (e.g., hepatitis B virus infection for liver cancer), the magnitude and pattern of increased risks substantially vary according to individual and clinical characteristics and to the modality of RRT [4].

It is well known that emerging techniques and advances in dialysis technology have led to significant improvements in patients’ life span. Due to increased survival, malignant neoplasms have become an increasingly relevant health issue in the dialysis population [5]. Previous studies have provided convincing evidence of an increased risk of certain cancer types, such as kidney, thyroid, and bladder cancer [1, 3, 6, 7], possibly related to side effects of kidney failure, including the prolonged uremic state, the presence of chronic infection and inflammation, a weakened immune system, nutritional deficiencies, and impaired mechanisms of DNA repair [8].

The investigation of the pattern of cancer risk in dialysis patients is crucial for planning primary and secondary cancer prevention strategies. The increased risk of cancer in dialysis patients has been well documented in Asia [3, 6], United States [7], Australia/New Zealand [2], and northern Europe [9]. Conversely, less evidence has been accumulated in southern European countries, such as Italy, where end-stage kidney disease (ESKD) patients represent about 0.1% of the general population [10], and only a small study has been published in the international literature [11].

In this population-based investigation, we assessed the risk and spectrum of de novo malignancies (DNMs) among dialysis patients as compared to the corresponding general population.

## Methods

A population-based cohort study was conducted using information ascertained through a record linkage procedure from three health-related databases, which cover the totality of the population in the Friuli Venezia Giulia region (1,227,000 inhabitants): 1) the health information system, which provides personal (e.g., sex, birthdate, residence, and vital status) and medical care data (e.g., hospital discharge, outpatient care, and pharmaceutical health care); 2) the renal registry database, which includes information about patients who underwent at least two dialytic sessions (either peritoneal dialysis or hemodialysis) per week for at least 90 days; 3) the population-based cancer registry, which collects data on all new cases of cancer occurring among the population living in the Friuli Venezia Giulia region. The cancer registry started collecting data on new cancer diagnoses in the resident population in January 1995. For the aims of this analysis, and to guarantee the highest completeness and accuracy of information, data from these three databases were linked only to the concurrently available period, i.e., 1998–2013.

For the aim of this study, the analysis was restricted to dialysis patients residing in the Friuli Venezia Giulia region between 1998 and 2013 and aged 40 years or older at their first dialysis. We focused on dialysis patients aged ≥40 years, because this age group covered 95% of all patients who had started their first dialysis between 1998 and 2013 and 99% of all cancer diagnoses in this population. Excluded from the analysis were subjects who met at least one of the following conditions: (i) KT before dialysis (*N* = 709); (ii) residence outside the Friuli Venezia Giulia region during the dialysis, or missing information on residence (*N* = 113); (iii) follow-up shorter than 30 days (*N* = 22). Accordingly to these criteria, 3407 dialysis patients represented our study population.

Cancer diagnoses were coded according to the International Classification of Disease, 9th revision (ICD-9). Cancer diagnoses done by autopsy only (i.e., post-mortem diagnoses; *n* = 12) were not considered in this study because of lack of completeness of this information during the whole registration period. Multiple primary tumours were included in the site-specific analyses, while for patients diagnosed with more than one DNM within the same ICD-9 group (e.g., colon, rectum, and anus ICD-9 codes: 153–154), only the first one was considered.

Person-years at risk were calculated from 30 days after the date of first dialysis to the date of DNM diagnosis, to the date of KT, to the date of death, to end of follow-up or to December 31, 2013 (i.e., censored cases), whichever came first. We excluded from the analysis the first 30 days of follow-up in order to reduce prevalent DNMs. After a DNM diagnosis, patients did not contribute any longer to person-time at risk for that specific cancer site/type. Conversely, those patients continued to be at risk of other cancer sites/types in the specific analyses. Patients with a history of cancer prior to their first dialysis were not considered eligible to contribute to person-time at risk for that specific cancer site/type.

The cumulative cancer incidence by time since first dialysis and cancer site/type was estimated using a competing risk approach with nonparametric estimators [12], taking into account death as a competing event. Standardized incidence ratios (SIRs) were estimated as the ratio between the observed number of cancer cases among the 3407 cohort members and the expected number of cancer cases among the general population of the Friuli Venezia Giulia region. According to a standard procedure [13–15], the expected number of cases was computed by multiplying the person-years at risk among the 3407 dialysis patients with the sex- and age-adjusted cancer incidence rates in the general population of the Friuli Venezia Giulia region derived from the local population-based cancer registry. Incidence rates for the general population were computed using, as numerator, the observed number of cancer cases, and, as denominator, the average resident population of same sex and age, as a proxy of person-years at risk.

SIRs for all cancers combined were also estimated according to sex, age at first dialysis (40–59, 60–74, ≥75 years), calendar period at first dialysis (1998–2001, 2002–2005, 2006–2009, 2010–2013), follow-up time (< 1, 1 to < 2, 2 to < 3, 3 to < 5, ≥5 years), and dialysis modality (haemodialysis, peritoneal dialysis, both haemodialysis and peritoneal dialysis). Corresponding 95% confidence intervals (CIs) for SIRs were computed assuming a Poisson distribution [13].

## Results

The 3407 dialysis patients were followed up for a total of 10,798 person-years (Table 1), with a median follow-up time of 2.3 years (interquartile range, IQR: 1.0–4.5 years). Most of these 3407 patients were males (62.4%), aged between 60 and 74 years at first dialysis (42.6%; median age: 70 years; IQR: 61–77 years), and were treated with haemodialysis (83.9%). More than one third underwent dialysis between 1998 and 2001 (35.5%). During the period of observation, 357 DNMs were diagnosed in 330 dialysis patients (9.7%) of whom, 25 had more than one DNM.

**Table 1:** Distribution of 3407 dialysis patients by selected characteristics. Friuli Venezia Giulia, north-eastern Italy, 1998–2013

	All patients
	No. (%)
Sex	
Male	2126 (62.4)
Female	1281 (37.6)
Age at first dialysis (years)	
40–59	746 (21.9)
60–74	1452 (42.6)
≥ 75	1209 (35.5)
Median (Interquartile Range)	70 (61–77)
Calendar year at first dialysis	
1998–2001	1208 (35.5)
2002–2005	674 (19.8)
2006–2009	802 (23.5)
2010–2013	723 (21.2)
Dialysis modality	
Haemodialysis	2858 (83.9)
Peritoneal dialysis	201 (5.9)
Both haemodialysis and peritoneal dialysis	348 (10.2)
Follow-up (years)	
< 1	867 (25.4)
1- < 2	674 (19.8)
2- < 3	539 (15.8)
3- < 5	632 (18.6)
≥ 5	695 (20.4)
Median (Interquartile Range)	2.3 (1.0–4.5)
Total person-years	10,798
Patient with de novo malignancies during dialysis	
No	3077 (90.3)
Yes	330 (9.7)
*No. of* de novo *malignancies during dialysis*	
*1*	*305 (92.4)*
*2*	*23 (7.0)*
*3*	*2 (0.6)*

**Fig. 1:**
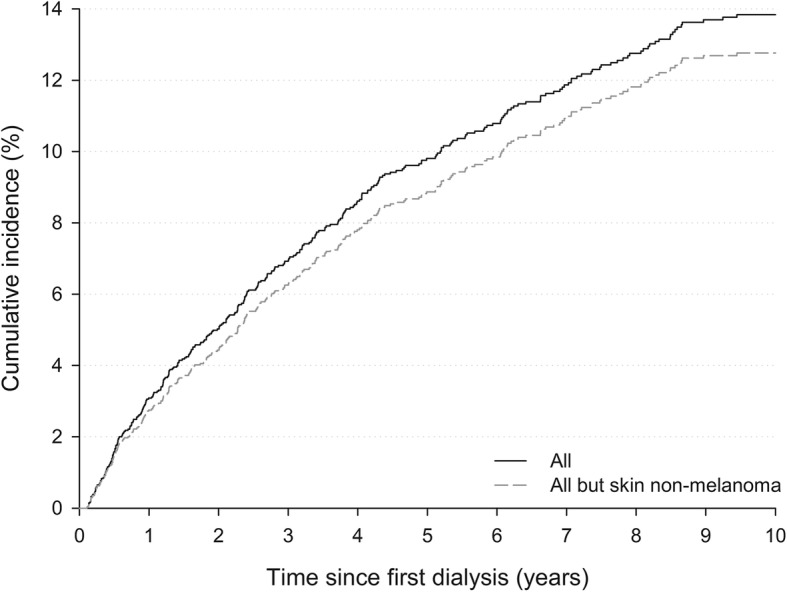
Cumulative cancer incidence by time since first dialysis. Friuli Venezia Giulia, north-eastern Italy, 1998–2013

The cumulative incidence of all DNMs combined steadily increased with time since beginning of dialysis, reaching 9.8% after 5 years and 13.9% after 10 years (Fig. 1). A similar pattern emerged after the exclusion of non-melanoma skin cancers (NMSC), with 5- and 10-year cumulative risks equal to 8.9% and 12.8%, respectively.

**Fig. 2:**
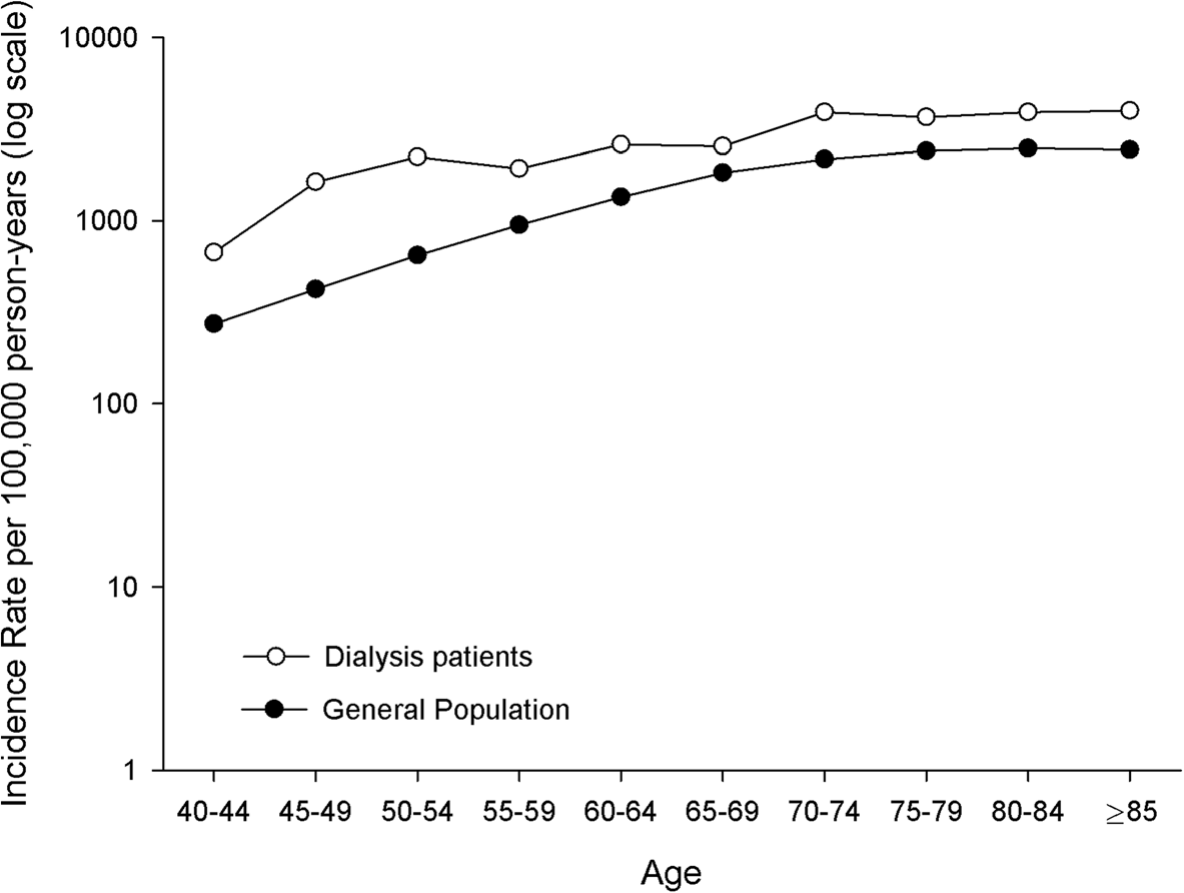
Age-specific incidence rates for de novo malignancies observed in dialysis patients and in the general population of Friuli Venezia Giulia region. Friuli Venezia Giulia, north-eastern Italy, 1998–2013

Starting at 40–44 years, the age-specific incidence rates for de novo malignancies observed among dialysis patients steadily increased from 670 per 100,000 PYs to 3965 per 100,000 PYs among those aged 85 years and older (Fig. 2). In dialysis patients, incidence rates were consistently higher than those in the general population of the Friuli Venezia Giulia region, even though these differences decreased with increasing age.

Considering all DNMs combined, a 1.3-fold higher risk was found in dialysis patients than in the corresponding general population (95% CI: 1.15–1.43) (Table 2). The subgroup analysis showed similar SIRs for both sexes (SIR = 1.29, 95% CI: 1.14–1.46 in males and SIR = 1.27, 95% CI: 1.01–1.57 in females). Furthermore, the risk of DNM was particularly high in younger dialysis patients (SIR = 1.88, 95% CI: 1.42–2.45 for age 40–59 years) and decreased with age. Elevated excess risks were also observed during the first 3 years since dialysis initiation, especially within the first year (SIR = 8.52, 95% CI: 6.89–10.41). When comparing the risk of DNM according to the modality of dialysis, the SIRs were 1.26 (95% CI: 1.12–1.42) in patients treated with haemodialysis and 1.78 (95% CI: 0.95–3.04; based on 13 observed cases of DNMs) in those treated with peritoneal dialysis.

**Table 2:** Standardized incidence ratios (SIRs) and 95% confidence intervals (CIs) in dialysis patients for all de novo malignancies (DNMs), by selected subgroups. Friuli Venezia Giulia, north-eastern Italy, 1998–2013

	Total^a^		
	Obs.	Exp.	SIR (95% CI)
All	330	256.6	1.29 (1.15–1.43)
Sex			
Male	246	190.5	1.29 (1.14–1.46)
Female	84	66.2	1.27 (1.01–1.57)
Age at first dialysis (years)			
40–59	56	29.7	1.88 (1.42–2.45)
60–74	176	131.0	1.34 (1.15–1.57)
≥ 75	98	95.9	1.02 (0.83–1.24)
Calendar year at first dialysis			
1998–2001	136	106.2	1.28 (1.07–1.52)
2002–2005	79	63.3	1.25 (0.99–1.56)
2006–2009	76	60.6	1.25 (0.99–1.58)
2010–2013	39	26.6	1.47 (1.04–2.00)
Dialysis modality			
Haemodialysis	279	221.7	1.26 (1.12–1.42)
Peritoneal dialysis	13	7.3	1.78 (0.95–3.04)
Both haemodialysis and peritoneal dialysis	38	27.7	1.37 (0.97–1.88)
Follow-up (years)			
< 1	95	11.2	8.52 (6.89–10.41)
1- < 2	52	24.2	2.15 (1.61–2.82)
2- < 3	48	32.2	1.49 (1.10–1.97)
3- < 5	56	62.0	0.90 (0.68–1.17)
≥ 5	79	127.1	0.62 (0.49–0.77)

^a^For patients diagnosed with more than one DNM, only the first one was considered; Obs., observed number of cancer cases; Exp., expected number of cancer cases

Table 3 lists the SIRs for specific DNMs with at least two observed cases. When considering all cancer types/sites other than NMSC, a 1.2-fold elevated cancer risk emerged, as compared to the general population (95% CI:1.03–1.32). Statistically significant increased risks were documented for NMSC (SIR = 1.81; 95% CI: 1.46–2.22), cancers of kidney (SIR = 3.18; 95% CI: 2.06–4.69), oral cavity (SIR = 2.42, 95% CI: 1.36–4.00), and Kaposi’s sarcoma (SIR = 10.29, 95% CI: 1.25–37.16).

**Table 3:** Standardized incidence ratios (SIRs) and 95% confidence intervals (CIs) in dialysis patients for selected de novo malignancies (DNMs). Friuli Venezia Giulia, north-eastern Italy, 1998–2013

		Total^a^		
Cancer type/site	ICD-9 codes	Obs.	Exp.	SIR (95% CI)
All but skin non-melanoma^b^		249	213.7	1.17 (1.03–1.32)
Skin non-melanoma	173	93	51.3	1.81 (1.46–2.22)
Prostate	185	35	39.5	0.89 (0.62–1.23)
Trachea, bronchus, and lung	162	31	28.4	1.09 (0.74–1.55)
Colon, rectum, and anus	153, 154	27	31.7	0.85 (0.56–1.24)
Kidney	189	25	7.9	3.18 (2.06–4.69)
Bladder	188	16	17.0	0.94 (0.54–1.52)
Oral cavity	140–149	15	6.2	2.42 (1.36–4.00)
Stomach	151	14	11.6	1.20 (0.66–2.02)
Liver	155	14	9.2	1.53 (0.83–2.56)
Female breast	174	13	14.9	0.81 (0.42–1.41)
Melanoma	172	9	5	1.81 (0.83–3.43)
Pancreas	157	7	7.5	0.93 (0.38–1.93)
Larynx	161	7	3.4	2.03 (0.82–4.18)
Other and ill-defined sites	195	7	1	7.31 (2.94–15.07)
Malignant neoplasm without site specification	199	7	3.8	1.86 (0.75–3.84)
Multiple myeloma	203	5	2.9	1.72 (0.56–4.02)
Leukemia	204–208	4	4.2	0.95 (0.26–2.43)
Corpus uteri	182	4	2.1	1.94 (0.53–4.96)
Pleura	163	2	1.8	1.11 (0.13–4.00)
Other and ill-defined sites within the respiratory system	165	2	0.6	3.14 (0.38–11.33)
Connective and other soft tissue	171	2	1.1	1.90 (0.23–6.87)
Kaposi’s sarcoma	176	2	0.2	10.29 (1.25–37.16)
Uterus, unspecified	179	2	0.3	7.0 (0.85–25.30)
Thyroid gland	193	2	1.4	1.39 (0.17–5.01)
Non-Hodgkin lymphoma	200,202	2	6.6	0.30 (0.04–1.10)

^a^The sums can exceed the total because some patients were diagnosed with more than one malignancy. For patients diagnosed with more than one DNM within the same ICD-9 group (e.g., oral cavity ICD-9 codes: 140–149; leukemia ICD-9 codes: 204–208) only the first one was considered; ^b^It includes sites/types with < 2 observed cases, which were not shown in table; Obs., observed number of cancer cases; Exp., expected number of cancer cases

## Discussion

The findings from this population-based cohort study conducted in north-eastern Italy showed that the cumulative cancer risk of dialysis patients reached about 14% after 10 years of follow-up. This observation corresponded to an overall 1.3-fold higher risk of DNM, as compared to the general population. Significantly increased cancer risks were seen for younger patients, and within the first three years of dialysis, as well as for several cancer sites/types, including kidney, oral cavity, NMSC, and Kaposi’s sarcoma.

Several studies have looked at the relationship between dialysis and cancer incidence, yielding overall estimates of excess cancer risk that were super-imposable to those reported in the present investigation [2, 3, 14]. Notably, results from a recent meta-analysis of population-based cohort studies showed that, in comparison with the general population, the pooled SIRs for DNM in dialysis patients including or excluding NMSC were 1.40 (95% CI: 1.36–1.45) and 1.35 (95% CI: 1.23–1.50), respectively [16]. To the best of our knowledge, only one study, carried out in Denmark, reported no evidence of a statistically significant increased risk of cancer after excluding NMSC (SIR = 1.16, 95% CI: 0.92–1.45) [15].

In the subgroup analyses, we found increased cancer risks that were in line with previous studies carried out in other industrialized countries [1, 3, 17]. Our results showed that the excess risk of cancer was higher in younger dialysis patients, while it declined with increasing age up to 75 years. Accordingly, a multicenter cohort study reported a high excess risk in the youngest age group (i.e., < 45 years at dialysis), which became lower in patients older than 65 years [14]. This age-related pattern may have different explanations. Firstly, the enhanced risk in younger patients is largely attributable to the low cancer incidence rates among young people in the general population. Secondly, younger patients may be affected by more serious virus-related malignancies against which their immune defenses, already compromised because of the uremic state, tend to be lacking, compared to older people [3, 18]. Thirdly, the presence of ESKD, comorbidities and frailty could lead elderly dialysis patients to die from other chronic illnesses, such as cardiovascular diseases, before the development of cancer. Thus, the discrepancy in cancer risk may disappear with increasing age.

We observed an elevated cancer risk among both male and female patients. A large international collaborative study reported that the overall risk for cancer was higher in females than in males during dialysis [1], but other investigations have reported contradictory findings [19].

Subgroup analysis by follow-up time showed that the risk of DNM was highest within the first year after dialysis initiation and decreased proportionally with the duration of dialysis. Similar figures were found in other investigations [1, 3, 17], with the exception of a large-scale cohort study, which reported the highest cancer risk after 4 years following dialysis initiation [5]. The increased risk of cancer in the first year of dialysis may be partially explained by presence of malignancies, without clinical signs, prior to RRT. Indeed, closer medical surveillance of chronic dialysis patients may have resulted in greater detection of tumors.

The analysis according to dialysis modality was limited because of the small number of DNMs in patients receiving peritoneal dialysis (i.e., *n* = 13 DNMs). Nonetheless, we documented a statistically significant increased risk of cancer in patients receiving haemodialysis, and an excess risk of borderline statistical significance in the remaining patients. To the best of our knowledge, only one recent investigation compared the two groups directly, showing no significant differences in cancer risk [20].

The present study also highlighted a strongly elevated risk of site-specific cancers. In accordance with other studies [1–3, 14, 17], a high risk was observed for kidney cancer, probably attributable to the nature of chronic kidney disease (CKD), related urological anomalies or the occurrence of acquired cystic kidney disease [21]. In contrast with other studies [16], we did not find a high risk of bladder cancer, which is strongly associated to several diseases that cause ESKD.

Our estimate of Kaposi’s sarcoma risk was similar to the findings from a large population-based cohort study [22]. The elevated excess risk emerged for Kaposi’s sarcoma is consistent with the evidence that the uremic immune dysfunction status of dialysis patients exposes them to be susceptible to viruses, such as Kaposi’s sarcoma-associated herpes virus (KSHV). Moreover, the prevalence of KSHV has been reported to be higher in some Mediterranean countries, particularly in Italy [23, 24]. Accordingly, the high excess risk found for cancers of oral cavity provided further support to the poor immune control of known oncogenic viruses in the ESKD population. Indeed, previous studies have shown higher risks of tongue or mouth cancers [22], which are known to be associated with human papillomavirus. Unfortunately, due to the low number of observed cases in the present investigation, we could not evaluate the SIRs for specific oral cavity subtypes.

NMSC was the most common DNM diagnosed among dialysis patients. Although only a few studies have investigated NMSC during dialysis, our findings confirmed prior evidence [25]. A recent Asian study reported that dialysis patients with uremic pruritus carried a higher risk of NMSC than those without, suggesting that the synergic effect of sun exposure and uremia could be responsible for the carcinogenesis of NMSC [25].

The advanced CKD and side effects of decreased renal function, including impaired function of the immune system and of DNA repair mechanisms as well as chronic infections and inflammations, have been proposed as potential promoting factors of cancer [22, 26]. To this regard, the higher risks found in the present study are consistent with the evidence that direct carcinogenic effects of factors related to ESKD are mediated by dialysis treatments [2]. Previous studies have provided limited support to the fact that dialysis may itself increase the risk of cancer [4]. However, it may prolong pre-RRT carcinogenic exposure since it neither completely replaces renal function nor reverses kidney disease [2].

Some limitations of our study are worth noticing. First, as for most studies [16], information about patients’ comorbidities -which are highly prevalent in this population- as well as information about etiology for ESKD or lifestyle habits (e.g., smoking, alcohol consumption, or sun exposure) are not routinely collected in the renal registry of the Friuli Venezia Giulia region. Consequently, we could not evaluate their potential confounding effect on the occurrence of cancer in dialysis population. However, this lack of information had limited impact on our aim of quantifying the excess risk of cancer among dialysis patients, in comparison with the corresponding general population. Second, we could not exclude the possibility of overestimation of cancer incidence, as a result of misclassification of prevalent cases as incident ones. However, to minimize this problem we excluded from the analysis the first 30 days of dialysis. A longer lag-time could have been considered between the occurrence and diagnosis of cancer, as we observed the highest excess risk of cancer within the first year. Nevertheless, a sensitivity analysis performed excluding the first 90 days of follow-up showed similar results. Third, the relatively small size of our cohort limited the study power to assess the excess risk of cancer for specific cancer sites/types. Finally, subgroup analysis should be interpreted with caution as the general population cannot be stratified by age at first dialysis, time on dialysis, dialysis modality, and calendar period at first dialysis. As most studies [16], the expected number of cases in each subgroup was calculated according to the incidence rates computed among the average population of Friuli Venezia Giulia region used for the non-stratified analysis. Notwithstanding such limitations, this population-based study represents the largest cohort to provide an overall picture of cancer risk among dialysis patients in southern Europe. One of the strengths of this study is the complete coverage of Friuli Venezia Giulia dialysis patients thanks to the use of population-based administrative data, which allows a comprehensive assessment of the health status of the whole population of the Friuli Venezia Giulia region. In addition, the use of incidence data collected by the same population-based cancer registry, ensures high standards in terms of quality and completeness of cancer reporting.

## Conclusions

Our findings put in evidence the need of monitoring the cancer burden among ESKD patients undergoing dialysis. The increased risks documented in relation to specific cancer sites and in younger patients suggest that targeted approach to cancer screening need to be implemented in the dialysis population. The link between renal function and cancer risk, and the detection of possible mechanisms through which renal impairment can modulate cancer risk remain a topic of great scientific interest.

## Abbreviations

CI: Confidence interval
CKD: Chronic kidney disease
DNM: De novo malignancy
ESKD: End-stage kidney disease
ICD-9: International Classification of Disease, 9th revision
IQR: Interquartile range
KSHV: Kaposi’s sarcoma-associated herpes virus
KT: Kidney transplant
NMSC: Non-melanoma skin cancers
RRT: Renal replacement therapies
SIR: Standardized incidence ratio
